# Managing Communication Challenges in Mental Health Care by Using Mobile Translation Apps: Protocol for a Simulation-Based Pilot Study

**DOI:** 10.2196/86787

**Published:** 2026-06-19

**Authors:** Annika Kreienbrinck, Saskia Hanft-Robert, Muhammed-Talha Topçu, Mike Mösko

**Affiliations:** 1 Department of Medical Psychology University Medical Center Hamburg-Eppendorf Hamburg Germany; 2 Department of Applied Human Sciences Hochschule Magdeburg-Stendal Stendal Germany; 3 Division of Health Systems and Public Health Faculty of Medicine and Health Sciences Stellenbosch University Stellenbosch, Western Cape South Africa

**Keywords:** language barriers, mental health care, translation tools, machine translation, app, feasibility, simulation, pilot study, multilingual care

## Abstract

**Background:**

Language barriers in mental health care can hinder diagnostic accuracy, communication quality, and therapeutic rapport. Multilingual mental health care providers (MHCPs) or qualified interpreters are not always available, prompting interest in mobile translation apps as alternative tools. The usability of such tools in mental health consultations remains underexplored.

**Objective:**

This pilot study aims to assess whether a mobile machine translation app can support MHCPs in conducting accurate mental health assessments with patients who speak a different language. Secondary objectives include assessing usability, communication quality, translation accuracy, and nonverbal interaction in a simulated mental health care setting. The feasibility objectives are to examine recruitment, retention, data completeness, time adherence, acceptability, scenario realism, and resource requirements to inform the design of a larger future trial.

**Methods:**

This is a single-arm, simulation-based, mixed methods pilot study involving 12 German-speaking MHCPs and 2 Turkish-speaking standardized service users. In this study, MHCPs include both fully trained professionals and individuals in advanced training involved in mental health care delivery. Each health care provider will participate in a simulated mental health consultation lasting up to 30 minutes, using the mobile translation app Mabel, which provides real-time translation during multilingual clinical interactions. Data will be collected via video and audio recordings, app use metrics, postsimulation questionnaires, and optional qualitative interviews. Data will be analyzed using descriptive statistics, thematic analysis, and mixed methods triangulation.

**Results:**

As of March 2026, no participants have been enrolled. The pilot study is being conducted within an ongoing Volkswagen Foundation-funded research project. Preparatory work, including scenario development, scoring rubrics, and data collection materials, is at an advanced stage. Recruitment is scheduled to begin in May 2026, with data collection completion anticipated in July 2026. Results will be reported in a future publication.

**Conclusions:**

This pilot study will provide initial evidence on the usability and feasibility of mobile translation apps in mental health care and inform the design of a larger implementation study.

**Trial Registration:**

OSF Registries osf.io/7r2hn; https://osf.io/7r2hn

**International Registered Report Identifier (IRRID):**

PRR1-10.2196/86787

## Introduction

### Background

Language barriers are a persistent challenge in health care systems worldwide, influencing access to services, diagnostic accuracy, treatment adherence, and overall quality of care [1-4]. These issues are especially pronounced in mental health care, where verbal communication is central to understanding service users’ (SUs) experiences, symptoms, and needs [5,6]. When health care providers and SUs do not share a common language, it can lead to miscommunication, incomplete clinical assessments, and a weakened therapeutic alliance, often resulting in premature discontinuation of care [3,7,8].

While qualified interpreters are widely recognized as the preferred method for overcoming language barriers, their availability is often limited by logistical, financial, and legal constraints [9-11]. Consequently, health care professionals frequently rely on informal or ad hoc strategies such as interpreting by family members, the use of multilingual staff, or gesturing and “receptive multilingualism” [10,12,13]. Although sometimes practical, these approaches can introduce risks, such as misinterpretation, omissions of clinically relevant information, and breaches of confidentiality [3,13,14].

In recent years, technological tools, particularly mobile translation apps, have gained attention as potential solutions to bridge communication gaps in the absence of qualified interpreters. These tools include machine translation apps and fixed-phrase tools specifically designed for health care environments [15-20]. Mobile translation apps have been proposed as scalable, cost-effective, and potentially real-time solutions for supporting basic communication, particularly in time-sensitive or resource-limited contexts; however, concerns remain regarding their translation accuracy, contextual appropriateness, and suitability for complex clinical interactions [16,21-24]. Emerging evidence suggests that mobile translation apps are already beginning to enter multilingual health care practice, often through informal and individual use by health care providers or SUs when professional interpreting services or other solutions are not readily available [22,25].

Findings from a recent scoping review identified 16 mobile translation tools used in health care settings and emphasized the heterogeneity of their usability and performance [16]. Most of the included studies were conducted in general medical or emergency care contexts. While some tools were rated positively for user satisfaction and ease of use, challenges persisted with translation accuracy, limited language coverage, and a lack of insight into the tools’ ability to handle complex or emotionally sensitive interactions [16-18,26]. Notably, the review found that very few studies have systematically explored how such tools function in psychosocial and mental health consultations, despite growing demand in multilingual mental health care environments [16].

Given these gaps, there is a pressing need to evaluate whether mobile translation apps can facilitate meaningful and accurate clinical communication in mental health care. Simulation-based studies provide a controlled, replicable, and ethically sound approach to examining human-technology interactions, particularly in scenarios where real-world implementation may pose clinical risks. This study aims to contribute to the evidence base by piloting a simulation approach that explores the diagnostic accuracy, communication quality, and usability of mobile translation tools in mental health consultations.

### Objectives

#### Primary Objective

The primary objective is to explore whether mental health care professionals are able to identify and assess mental health conditions when communication is mediated through a mobile translation app in simulated SU interactions involving a language barrier.

#### Secondary Objectives

The secondary objectives of this study are as follows:

- To assess the usability of a mobile translation app in a simulated clinical mental health setting
- To evaluate the quality of communication and rapport established between mental health care providers (MHCPs) and standardized SUs
- To examine the accuracy of translations produced by the mobile app, with particular focus on clinically relevant error types
- To explore nonverbal communication behaviors during translated mental health interactions

#### Feasibility Objectives

The feasibility objectives of this study are as follows:

- To assess recruitment feasibility, including the proportion of eligible participants enrolled within the planned time frame
- To evaluate retention and completion of the full simulation protocol
- To examine data completeness and quality across video and audio recordings, transcripts, and questionnaires
- To assess adherence to the allocated consultation time
- To evaluate the acceptability of the simulation design and mobile translation app to participants
- To explore participants’ perceptions of the realism and plausibility of the simulation scenario
- To document resource requirements (time, personnel, and equipment) for study implementation

## Methods

### Study Design

This is a single-arm, simulation-based, mixed methods pilot study. Each simulation involves a one-on-one consultation between a German-speaking MHCP and a standardized SU who speaks only Turkish. Communication is facilitated via a mobile translation app. The aim is to evaluate the feasibility, usability, and quality of health care provider-SU interaction under these conditions in a controlled, simulated mental health consultation.

The study combines the following components:

- Quantitative data: standardized assessments of diagnostic accuracy, usability scores, and communication quality
- Qualitative data: observational field notes, open-ended responses, and optional interviews
- Mixed methods integration: triangulation of quantitative and qualitative findings

### Study Setting

The study will take place in a simulated outpatient consultation room, designed to replicate a real-world general practitioner or mental health care setting closely. Simulations will be conducted on-site at a medical training facility or university-based simulation laboratory.

### Eligibility Criteria

#### MHCP Participants

Eligible participants will include individuals involved in the provision of mental health care, including advanced-level students (eg, medical students after the first state examination or those enrolled in a clinical mental health program) and fully trained mental health care professionals. For this study, MHCPs are defined as individuals either in training or practice who engage in clinical mental health consultations. Individuals with proficiency in the Turkish language will be excluded.

#### Standardized SUs

Eligible standardized SUs will be trained actors or students portraying a SU with a mental health condition. They will be native Turkish speakers, trained in presenting scripted cases consistently.

### Interventions and Simulation Task

Each MHCP participates in 1 simulated consultation lasting up to 30 minutes, which reflects typical constraints in outpatient mental health care and allows for standardization across sessions. The case involves a new SU presenting with mental health symptoms (eg, major depressive disorder). The SU communicates exclusively in Turkish. No human interpreter will be present.

The simulation scenario and script will be developed collaboratively by the research team in consultation with practicing MHCPs and, where possible, with input from individuals with lived experience of mental health conditions and migration. The case will be based on typical presentations of major depressive disorder in primary or outpatient mental health settings and adapted for cultural and linguistic plausibility in the German-Turkish context. The scenario will be pretested during preparatory training with the standardized SU and revised, if necessary, to optimize clarity, realism, and consistency across simulations.

Communication during the simulation will be facilitated using the mobile translation app Mabel (Mabel AI) [27]. Mabel is a digital translation tool designed to support multilingual communication in health care settings by providing real-time voice-based translation during conversations. The app also allows the generation of conversation transcripts, which can support later review and analysis. The tool was selected based on several predefined criteria, including its suitability for health care communication, the availability of real-time translation features, and compliance with relevant data protection standards. According to the app developer, the system is compliant with both the General Data Protection Regulation (GDPR) and the Health Insurance Portability and Accountability Act. The app will be used on a smartphone provided for the simulation sessions, and participants will receive a brief introduction to its basic functionality before the consultation begins.

During the consultation, MHCPs will be instructed to perform a clinical assessment, formulate a preliminary diagnosis, and suggest next steps for care planning. They may use the app at their discretion.

### Outcomes

#### Primary Outcome

The primary outcome is diagnostic accuracy, assessed by comparing the MHCP’s diagnosis and clinical reasoning during the consultation to a reference diagnosis predefined by the research team based on the scenario script. Given the exploratory nature of this pilot study and the limited sample size, diagnostic accuracy will be interpreted as a preliminary indicator rather than a definitive measure of diagnostic performance. Each diagnosis will be scored using a structured rubric inspired by objective structured clinical examination formats commonly used in clinical skills assessment [28]. The diagnostic scoring rubric will assess three key components of the clinical assessment: (1) identification of the primary mental health condition presented in the scenario or relevant differential diagnoses, (2) recognition of key symptom domains described by the SU, and (3) the appropriateness of initial clinical reasoning and proposed next steps for care. Each component will be scored on a 3-point scale (0=incorrect or absent, 1=partially correct, and 2=correct), resulting in a total possible score ranging from 0 to 6. On the basis of the total score, diagnostic assessments will be categorized as accurate (5-6 points), partially accurate (3-4 points), or inaccurate (0-2 points). This structured approach allows a transparent and standardized evaluation of diagnostic performance across participants. Scoring will be performed independently and in a blinded manner by 2 researchers. Discrepancies will be resolved through discussion or consultation with a third researcher. The scoring rubric will be developed and refined during the preparatory phase of the study using the scripted case scenario.

#### Secondary Outcomes

Secondary outcomes include several dimensions of communication, translation performance, and interaction quality.

Usability of the mobile translation app will be assessed using the System Usability Scale (SUS), a validated 10-item questionnaire that provides a global measure of users’ perceived usability of digital systems. SUS scores range from 0 to 100, with scores above 68 typically indicating above-average usability [29]. The SUS captures key dimensions, such as efficiency, learnability, and user satisfaction, reflecting how easily users can interact with the system and accomplish their tasks. It offers a robust and quick benchmark for assessing perceived usability. The SUS has also been applied in health care settings involving a digital translation tool, where it helped assess whether such tools were experienced as intuitive and practical in high-stress communication scenarios [30,31]. In addition to questionnaire-based usability measures, app use will be documented using, when available, system-generated information provided by the translation app. Core use metrics will include the number of translation interactions initiated during the consultation and the total duration of app use during the simulated encounter. These indicators provide an overview of how frequently and for how long the translation tool is used during the consultation. The app also generates a transcript of the translated interaction, which will be used alongside the audio recordings of the consultation to analyze translation output. Where available, additional use information (eg, types of translation input, such as voice or text) may be explored descriptively. These metrics will provide contextual information on how the translation tool was used during the simulated consultation.

Interaction quality will be assessed using the Communication Assessment Tool (CAT), a validated instrument designed to measure SUs’ perceptions of health care providers’ communication skills. The CAT consists of 15 items rated on a Likert scale and has been widely used to evaluate communication quality in health care encounters. In this study, the CAT will be completed by the standardized SU immediately following each simulation [32]. In simulation-based communication research, standardized patients are commonly used to evaluate interaction quality because they directly experience the consultation while maintaining consistency across scenarios. To complement the SU perspective, MHCPs will complete a customized postsimulation questionnaire designed to capture their clinical and interpersonal experiences using the mobile translation app. The questionnaire comprises Likert scale items to evaluate the clarity and effectiveness of communication, including how well health care providers could convey questions and explanations, the app’s ability to preserve the nuances of the SU’s responses, and the need for rephrasing due to translation issues. It also assesses health care providers’ confidence in diagnostic interpretation, such as the ability to gather clinically relevant information and assess the SU’s emotional state. Health care providers will reflect on their ability to establish rapport and empathy. To identify practical challenges, the questionnaire includes items on technical or workflow disruptions (eg, delays and interface issues) and their impact on the consultation process. Additionally, health care providers will rate the cognitive and emotional demands of using the app during consultations, such as mental effort and stress levels. Finally, health care providers will evaluate the app’s perceived utility, including its role in supporting thorough assessments and their willingness to recommend it for future use. By focusing on these dimensions, the questionnaire complements the SUS, and in part the CAT, offering a holistic view of both communication quality and the health care provider’s experience during app-mediated consultations.

Translation accuracy will be evaluated by analyzing transcripts of the simulated consultations alongside the corresponding translation output produced by the app. Errors will be coded using a structured error typology adapted from the study by Delfani et al [17], which was designed to assess machine translation in mental health care contexts. This typology included the following categories:

- Inaccuracies in mental health and medical terminology (eg, mistranslation of symptoms, diagnoses, or treatment terms)
- Syntactic and semantic errors (eg, incorrect sentence structure or word choice)
- Comprehensibility issues (eg, unclear or confusing translations)
- Fluency issues (eg, unnatural phrasing or readability problems)
- Clarity and coherence issues (eg, ambiguous or disjointed translations)
- Critical errors (eg, errors that could mislead health care providers or pose risks to SU safety)

For this study, the framework will further be refined to include errors specific to real-time, app-mediated communication, such as omission of clinically relevant cues (eg, pauses) or misinterpretations due to app interface limitations. Translation errors will be coded independently by 2 bilingual raters fluent in both German and Turkish who have experience in translation or linguistic analysis. The raters will review transcripts of the simulated consultations and classify errors according to the predefined error typology. Coding guidelines will be developed prior to analysis to ensure consistent application of the error typology across raters. To enhance analytic rigor, both raters will conduct the coding independently. Interrater reliability will be assessed using Cohen kappa (κ) or a comparable agreement statistic, depending on the final structure of the coded units. Discrepancies will be resolved through discussion between raters or consultation with a third bilingual reviewer.

Nonverbal communication behaviors will be assessed through audio and video recordings of the simulations. An observational framework will be used by 2 independent raters to code key behaviors, such as eye contact, gesturing, and turn-taking, which contribute to rapport and interaction flow. Table 1 provides an overview of all study outcomes, corresponding data sources, and measurement approaches.

**Table 1:** Overview of study outcomes, corresponding data sources, and measurement approaches.

Outcome	Data source	Measurement or instrument
Diagnostic accuracy	MHCP^a^ written diagnosis and audio-recorded consultation transcripts	Structured diagnostic scoring rubric inspired by objective structured clinical examination formats, applied by 2 independent blinded raters
Usability	MHCP self-report and app use data	System Usability Scale questionnaire and app use metrics
Communication quality	MHCP and standardized SU^b^ self-report	Communication Assessment Tool by SU and a customized questionnaire for MHCPs
Translation accuracy	Transcripts of interactions and app-generated translation output	Coding of translation errors (eg, omission, distortion, and mistranslation)
Nonverbal communication behavior	Audio and video recordings	Structured observational coding framework (eg, eye contact and gestures) by 2 independent raters

^a^MHCP: mental health care provider.

^b^SU: service user.

### Participant Timeline

The study will follow a standardized timeline for each participant, as outlined in Table 2.

The schedule of enrollment, interventions, and assessments is presented in the SPIRIT (Standard Protocol Items: Recommendations for Interventional Trial) table (Table 3). The corresponding checklist is provided as Multimedia Appendix 1.

**Table 2:** Simulation timeline.

Phase	Estimated duration (min)	Activities
Before simulation	10	Brief introduction, informed consent, and demographic form Presimulation briefing
Simulation session	Maximum 30	Full consultation with standardized SU^a^ using the translation app
After study	10-15	Completion of usability and communication quality questionnaires (MHCP^b^ and SU) and brief feedback Optional brief interview and/or debrief

^a^SU: service user.

^b^MHCP: mental health care provider.

**Table 3:** SPIRIT (Standard Protocol Items: Recommendations for Interventional Trial) schedule of enrollment, interventions, and assessments.

Time point	Enrollment	Simulation (day 0)	Postsimulation phase
Informed consent	✓		
Eligibility screening	✓		
Sociodemographic survey		✓^a^	
Simulation (consultation)		✓	
Translation app use		✓	
Diagnostic accuracy scoring			✓
Usability survey			✓
Communication quality survey			✓
Translation error coding			✓
Observational coding			✓
Optional interviews			✓

^a^Conducted before the simulation session.

Each MHCP participates in 1 simulation. Following the simulation, participants will complete postsimulation questionnaires assessing usability and communication quality. Optional brief interviews and a debriefing session will also be offered. Given the potentially sensitive nature of mental health consultations, participants will have the opportunity to reflect on their experiences during the debriefing session and to discuss any concerns arising from the simulation. The entire study session (briefing, simulation, and debrief) is expected to last approximately 60 to 80 minutes per participant.

### Sample Size

As a pilot study, the primary goal is to assess feasibility and inform the design of a larger trial. On the basis of prior simulation studies in health care communication research, a sample size of 12 MHCPs (each participating in 1 simulation session) and 2 standardized SUs (each portraying 1 scripted case in Turkish) will be used [18,33].

### Recruitment

#### MHCP Participants

Participants will be recruited from advanced-level medical and mental health training programs, including psychotherapy, psychiatric nursing, or medical students with clinical exposure to mental health. While recruitment of licensed professionals (eg, psychotherapists and psychiatrists) is intended, we anticipate that most participants will be trainees. All participants will be considered MHCPs for this study, as the consultation setting and case scenario focus specifically on mental health care. Flyers, email invitations, and announcements in relevant seminars will be used. Inclusion and exclusion criteria will be communicated. Recruitment is expected to take approximately 4 to 6 weeks, depending on participant availability.

#### Standardized SUs

Two native Turkish-speaking acting students or trained simulation patients will be recruited and instructed to perform the same scripted case scenario. Prior to the study, standardized SUs will participate in preparatory training sessions with the research team to familiarize themselves with the case narrative, symptom presentation, and expected responses to typical clinical questions. These training sessions will include rehearsal of the scenario and discussion of how to maintain a consistent portrayal of symptoms and behaviors across consultations. The case script will serve as the reference framework to support standardization across simulations. To minimize fatigue effects and maintain performance consistency, simulation sessions will be scheduled with breaks between consultations. Simulations will be observed and reviewed through the recording process by the research team to monitor adherence to the case script and maintain consistency across sessions.

### Data Collection

#### Data Sources

To enable triangulation and capture a comprehensive understanding of the simulation process, data will be collected from multiple sources. Each simulation will be audio- and video-recorded to allow detailed analysis of both verbal and nonverbal communication. In addition, app use metrics will be tracked, including the number of translation interactions and the total duration of use.

Following each simulation, both the MHCPs and the SUs will complete postsimulation questionnaires. These will include validated instruments, such as the SUS and the CAT, completed by the standardized SU to evaluate communication quality. Optional open-ended written reflections or postsimulation interviews with MHCPs will explore experiences with the app, perceived challenges, and emotional responses. These will be audio-recorded and thematically analyzed to complement questionnaire data. Transcripts of the translated interactions will also be generated and systematically coded to identify and classify translation errors, such as omissions, distortions, or mistranslations.

#### Data Management

All data will be handled following strict data protection protocols. Video and audio files will be securely stored on password-protected institutional servers. Transcripts will be fully anonymized before analysis, with all names, institutions, and other potentially identifying information removed or replaced. Data management will comply with the GDPR and local institutional policies. Access to raw data will be restricted to the core research team, and no identifiable data will be published or shared.

### Statistical Methods

#### Overview

This study follows an exploratory mixed methods analytic strategy appropriate for a pilot study design. Quantitative and qualitative data collected during the simulation sessions will be analyzed separately and subsequently integrated to explore patterns related to communication processes, usability, and translation performance when using a mobile translation app in simulated mental health consultations. Given the exploratory nature and small sample size of the study, analyses will primarily focus on descriptive patterns and preliminary insights rather than hypothesis testing.

For outcomes requiring structured scoring or coding procedures (eg, diagnostic accuracy scoring and translation error coding), ratings will be conducted independently by 2 trained raters. Interrater reliability will be assessed using appropriate agreement statistics depending on the measurement scale (eg, Cohen κ), and discrepancies will be resolved through discussion or consultation with a third researcher.

#### Quantitative Analysis

Quantitative data will be analyzed using descriptive statistics to summarize key variables, including diagnostic accuracy scores, SUS ratings, and app use data. Frequencies and distributions will also be examined for different types of translation errors and interaction ratings. Where appropriate, exploratory correlation analyses may be conducted to investigate potential relationships, such as between usability ratings and diagnostic accuracy.

#### Qualitative Analysis

Qualitative data from open-ended responses and observational field notes will be analyzed thematically, following a structured approach to identify key themes and patterns [34]. Translation errors will be coded using a predefined framework that includes categories such as omission, mistranslation, and distortion.

#### Mixed Methods Integration

Findings from the quantitative and qualitative components of the study will be integrated using a triangulation approach. Quantitative indicators, such as diagnostic accuracy scores, usability ratings, communication assessments, and app use metrics, will be examined alongside qualitative insights derived from observational coding and optional interview data. Where appropriate, joint displays will be used to compare findings across data sources and to explore relationships among communication quality, usability perceptions, translation performance, and diagnostic outcomes. This integrated analysis will provide exploratory insights into how communication processes, usability perceptions, and translation performance interact during simulated consultations. In particular, combining CAT ratings from standardized SUs with self-reported health care provider experiences may help illustrate different perspectives on communication dynamics during translated consultations.

#### Feasibility Indicators

As a pilot study, a key objective is to evaluate the feasibility of using a mobile translation app in simulated mental health consultations. These feasibility findings will inform the design and implementation of a larger future study. To this end, we will assess several feasibility indicators related to recruitment, data collection, implementation fidelity, acceptability, and resource needs.

We will examine the recruitment rate, defined as the proportion of eligible MHCPs and SUs successfully enrolled within the planned time frame. Retention and completion will be assessed based on the percentage of participants who complete the full simulation protocol, including all scheduled data collection steps.

Data completeness will be evaluated through internal checks to ensure that all audio and video recordings, transcripts, and questionnaire responses are available and usable for analysis. In parallel, time adherence will be monitored by observing whether simulation sessions remain within the allocated 30-minute consultation window.

To explore the acceptability of the simulation setup and mobile translation app, both MHCPs and SUs will complete postsimulation questionnaires. These will include Likert scale items addressing perceived emotional burden, willingness to participate in similar studies again, and the overall experience of using the app in a clinical interaction.

In addition, scenario realism will be assessed by asking participants to rate how well the simulation mirrored real-world clinical encounters, including the plausibility of the case and communication dynamics.

Resource requirements will be documented by tracking the time, equipment, and personnel needed for the SU training, app setup, data collection, and analysis. These data will support logistical and budgetary planning for a potential trial. Overall, these measures will help determine whether the simulation design is appropriate for future scaled-up studies. An overview of all feasibility indicators, along with corresponding data sources and instruments, is provided in Table 4.

**Table 4:** Feasibility indicators, corresponding data sources, and measurement methods.

Feasibility indicators	Data source	Measurement or instrument
Recruitment rate	Recruitment records	Number enrolled and number approached
Retention and completion	Study records	Percentage of participants completing the full simulation protocol
Data completeness	Internal audit	Availability of audio and video recordings, transcripts, and questionnaire data
Time adherence	Researcher observation	Whether the simulation was completed within the 30-min time limit
Acceptability	MHCP^a^ and SU^b^ postsimulation questionnaire	Likert scale items on emotional burden and willingness to participate
Scenario realism	MHCP and SU postsimulation questionnaire	Likert scale items on perceived realism and plausibility
Resource requirement	Research team logs	Time and personnel for SU training, app setup, transcriptions, and further study tasks

^a^MHCP: mental health care provider.

^b^SU: service user.

As this study is designed as a pilot feasibility study, feasibility indicators will primarily be interpreted descriptively rather than against strictly predefined benchmarks. However, indicative thresholds will be used to support the interpretation of feasibility outcomes. For example, recruitment of at least 70% of approached eligible participants, completion of the full simulation protocol by at least 80% of enrolled participants, and high completeness of questionnaire and recording data (≥90%) will be considered indicative of acceptable feasibility for proceeding to a larger study.

### Ethical Considerations

Ethics approval for this study was obtained from the Local Psychological Ethics Committee, University Medical Center Hamburg-Eppendorf, on August 28, 2025 (LPEK-0967) prior to recruitment and data collection. All participants will receive written and verbal information about the study, including its aims, procedures, data protection measures, and their rights as participants. Written informed consent will be obtained from all MHCPs and SUs.

Video and audio data will be anonymized and securely stored on password-protected servers. Personal identifiers will be removed during transcription. The study will comply with the GDPR and local institutional policies of the University Medical Center Hamburg-Eppendorf, Germany, regarding research with human participants.

### Protocol Amendments

Any important protocol amendments (eg, changes to eligibility criteria, outcomes, or analysis methods) will be communicated to the ethics committee and relevant institutional authorities and, where applicable, will be reported in future publications. Updated protocol versions will be documented with a version number and date, and major amendments will be reported in future publications or registry updates where applicable.

### Dissemination Plan

The results of this pilot study will be disseminated through peer-reviewed journal articles, conference presentations, and stakeholder briefings. The study will inform the design of a larger trial on the use of translation tools in mental health care.

## Results

As of March 2026, this protocol corresponds to version 1.4. The study has received ethics approval and funding, but participant recruitment has not yet commenced. Preparatory work is at an advanced stage, including the development of the simulation scenario, refinement of the diagnostic accuracy scoring rubric, and preparation of data collection instruments and recording procedures. The study team is preparing to recruit and train 2 standardized simulation patients for the Turkish-language scenario and has finalized logistical procedures for the simulation sessions.

Recruitment of MHCPs is planned to begin in May 2026, with data collection expected to be completed by July 2026. No results are available yet. The findings of this pilot study, including feasibility indicators, are expected to be reported in a subsequent publication after completion of data collection and analysis.

## Discussion

This pilot study will generate preliminary evidence on the usability of a mobile translation app in simulated mental health consultations involving a language barrier. Although translation technologies are increasingly used in clinical practice, little is known about how they perform in mental health contexts, where communication relies strongly on nuance, precision, and interpersonal cues. Using a standardized simulation design allows the controlled examination of diagnostic accuracy, communication quality, translation performance, and nonverbal interaction while avoiding risks associated with real-world clinical encounters. This study does not aim to replace qualified interpreting services but rather to explore the quality and practical use of such tools in situations where qualified interpreters are not available. In this context, such tools should be understood primarily as temporary communication aids rather than substitutes for professional interpreting services, particularly in complex mental health assessments where nuanced communication and clinical judgment are essential. The strengths and limitations of the study are provided in Textbox 1.

**Strengths and limitations of this study.**

**Strengths**

- This study represents one of the first pilot studies to simulate mental health consultations using a mobile translation app in a controlled setting.
- The mixed methods approach will allow for triangulated analysis of usability, diagnostic accuracy, communication experience, and translation performance.
- The simulation design provides a safe and standardized environment to evaluate technology use in language-discordant mental health care interactions.

**Limitations**

- The findings may not generalize beyond the German-Turkish language pair or the specific app used.
- The simulation-based interactions may not fully capture the complexity and emotional dynamics of real-world consultations.

This study is expected to contribute early insights into how mobile machine translation tools function in complex, interaction-rich mental health encounters and to identify practical considerations for a larger future trial. The mixed methods approach supports a comprehensive understanding of both user experience and interaction processes.

Several anticipated limitations should be considered. Simulation-based interactions cannot fully reproduce the depth and unpredictability of real clinical encounters. In addition, the study focuses on a single language pair (German-Turkish), which may limit the transferability of findings to other linguistic contexts. The study also evaluates a specific mobile translation app; translation performance may vary across different systems depending on factors such as underlying algorithms, update cycles, and the handling of specialized terminology, and findings may therefore not be directly transferable to other translation technologies. Furthermore, the small sample size and the likely inclusion of health care providers in training may influence communication strategies and usability perceptions. The 30-minute consultation limit may restrict the complexity of clinical dialogue that can emerge during the interaction. In addition, the use of a small number of standardized SUs may introduce variability in how the scenario is portrayed across sessions despite preparatory training and efforts to maintain consistency. However, these constraints are inherent features of a pilot design and will help refine procedures for a potential future full-scale study.

Overall, this protocol outlines a structured and feasible approach to explore technology-supported communication in mental health care. The findings will help clarify the potential role of mobile translation apps and guide the design of a subsequent study.

## Data Availability

Data sharing is not applicable to this paper as no datasets have been generated or analyzed yet.

## Appendix

Multimedia Appendix 1 SPIRIT (Standard Protocol Items: Recommendations for Interventional Trial) checklist.

## Abbreviations

CAT: Communication Assessment Tool
GDPR: General Data Protection Regulation
MHCP: mental health care provider
SPIRIT: Standard Protocol Items: Recommendations for Interventional Trial
SU: service user
SUS: System Usability Scale
