# Supplementary material for: Managing Communication Challenges in Mental Health Care by Using Mobile Translation Apps: Protocol for a Simulation-Based Pilot Study
Source: JMIR Res Protoc. 2026 Jun 19;15:e86787. doi: 10.2196/86787 (PMC13332371; doi:10.2196/86787)
Supplement: Multimedia Appendix 1 [file resprot_v15i1e86787_app1.pdf]

Additional file 1:

SPIRIT Checklist - Managing communication challenges in mental healthcare using mobile translation applications: A simulation-based pilot study protocol

SPIRIT Item	Description	Protocol Reference
1. Title	Descriptive title identifying the study design, population, and intervention.	Title page: “Managing communication challenges in mental healthcare using mobile translation applications: A simulation-based pilot study protocol”
2a. Trial identifier and registry name	Trial identifier and registry name. If not yet registered, name of intended registry	Study not subject to mandatory trial registration. The study was registered on Open Science Framework (https://doi.org/10.17605/OSF.IO/7R2HN).
2b. WHO Trial Registration Data Set	All items from the WHO Trial Registration Data Set.	Not applicable
3. Protocol version	Date and version identifier.	Protocol version: 1.3, March 2026 (Trial status section)
4. Funding	Sources and types of financial, material, and other support.	Funding: MiM2M project (Funding section)
5. Roles and responsibilities	Names, affiliations, and	Authors and affiliations listed on title page and Authors’ contributions section

	roles of protocol contributors.	
6a. Background and rationale	Description of research question and justification for undertaking the trial, including summary of relevant studies (published and unpublished) examining benefits and harms for each intervention	Background section
6b. Explanation for choice of comparators	Rationale for comparators.	Not applicable - single-arm pilot study
7. Objectives	Specific objectives or hypotheses.	Objectives section
8. Trial design	Description of trial design including type and allocation ratio.	Trial Design section: single-arm, simulation-based, mixed-methods pilot study.
9. Study setting	Description of study settings.	Methods - Study setting.
10. Eligibility criteria	Inclusion and exclusion criteria.	Methods - Eligibility criteria.

11. Interventions	Intervention details sufficient for replication.	Methods - Interventions / Simulation Task
12. Outcomes	Primary, secondary outcomes and how/when they are assessed.	Methods - Outcomes
13. Participant timeline	Schedule of enrolment, interventions, assessments.	Participant timeline section and Table 2
14. Sample size	Estimated number of participants and how it was determined.	Sample size section
15. Recruitment	How participants will be recruited.	Recruitment section
16. Allocation	Sequence generation, concealment mechanism, implementation.	Not applicable - single-arm study
17. Blinding (masking)	Who is blinded after assignment and how.	Not applicable - single-arm, open simulation study
18a. Data collection methods	Plans for assessment and collection of outcome data.	Methods - Data collection
18b. Data quality	Plans to promote data quality.	Video recording and standardised instruments mentioned in Data collection

19. Data management	Plans for data entry, coding, security, and storage.	Data management and security section
20a. Statistical methods	Statistical methods for analysing primary and secondary outcomes.	Statistical methods and analysis plan
20b. Additional analyses	Methods for any additional analyses (e.g., subgroup).	Exploratory correlation analysis - Quantitative analysis section
20c. Analysis population and missing data	Definition of analysis population, handling of missing data.	Pilot design: not explicitly detailed
21a. Data monitoring committee (DMC)	Composition and role of DMC.	Not applicable - minimal risk pilot study
21b. Interim analyses	Plans for interim analysis and stopping guidelines.	Not applicable
22. Harms	Plans for collecting, assessing, reporting adverse events.	Ethics and dissemination section - minimal risk simulation
23. Auditing	Frequency and procedures for	Not applicable

	auditing trial conduct.	
24. Research ethics approval	Plans for seeking research ethics committee approval.	Ethics and dissemination section
25. Protocol amendments	Plans for communicating protocol amendments.	Ethics and dissemination - Protocol amendments
26. Consent	Who will obtain consent and how.	Informed consent section
27. Confidentiality	How personal data will be protected.	Data management and security section
28. Declaration of interests	Financial and other competing interests.	Declarations - Competing interests
29. Access to data	Who will have access to the final dataset.	Data management and security section
30. Ancillary and post-trial care	Provisions for post-trial care.	Not applicable – simulation study
31. Dissemination policy	Plans for communicating trial results.	Dissemination plan section
32. Informed consent materials	Model consent form and other materials.	Not included in protocol – in preparation stage

33. Biological specimens	Plans for collection, evaluation of biological specimens.	Not applicable – no biological samples
